# Realization of the EU's Cohesion Policy in Health Care in the Visegrad Group Countries in the Perspective 2014-2020

**DOI:** 10.3389/fpubh.2020.00133

**Published:** 2020-04-23

**Authors:** Tomasz Holecki, Iwona Kowalska-Bobko, Aldona Fraczkiewicz-Wronka, Maria Wegrzyn

**Affiliations:** ^1^Department of Health Economics and Management, Faculty of Health Sciences in Bytom, Medical University of Silesia, Katowice, Poland; ^2^Jagiellonian University, Medical College, Faculty of Health Science, Krakow, Poland; ^3^University of Economic, Department of Public Management, Katowice, Poland; ^4^Department of Finance, Faculty of Economics and Finance, Wroclaw University of Economics and Business, Wroclaw, Poland

**Keywords:** european union, cohesion policy, european health policy, public management, health care

## Abstract

A key objective of the European Union is to strengthen regional cohesion by addressing development disparities, particularly by targeting less-favored regions (1). Initiatives related to leveling development differences in the field of health care are recognized as a one of priorities in the European Union. Therefore, when implementing cohesion policy, decisions have been made to mobilize structural funds for sectoral activities. The aim of this paper is to present the European Union's cohesion policy in the field of health care and to indicate the most important actions of the implemented programmes/projects in selected countries: Poland, the Czech Republic, the Slovak Republic and Hungary—the Visegrad Group–VG4—in the period of 2014–2020. Analysis covers programmes, funding sources, and activities undertaken in achieving cohesion policy objectives in health care in the VG4.

## Introduction

As a regulator defining the foundations of socio-economic growth, the European Union, in its strategic documents, identifies goals and provides legal and financial tools supporting their achievement (2). This is pursued through investment in innovative solutions in the service sector, including health care (3). Initiatives aimed at the elimination of developmental disparities come under the umbrella term—cohesion policy. As a result of legislative actions taken over many years, new instruments necessary to achieve the goals set out in cohesion policy have been developed and implemented. Additionally, priorities are set for every programming period.

The current programming perspective (2014–2020) identifies health as one of the most important areas requiring systemic aid. The European Union has prioritized initiatives ensuring decent conditions for achieving health and has taken decisions to provide structural funds for sectoral actions in health care, but it has also established conditions under which funds can be granted to individual member states and requires them to set up an institutional framework allowing for the adequate absorption of aid funds. The main condition is to plan and implement effective coordination of public expenditures funded by the European Union (4). The European Union, acting in its capacity as a regulator shaping the foundations of socio-economic development, focuses on supporting actions aimed at sustainable growth and harmonious economic activity, including initiatives to reduce developmental disparities in health. This is a consequence of acknowledging the following statement as true: not only does unequal access to health care services lead to deteriorating health in society, but it also generates measurable economic losses due to the lack of health and failure to undertake economic activity and remain active. In recent decades, technology-related costs and progress in treatment, as well as the aging population, have been identified as the most important factors contributing to increased health expenditure (5, 6).

Technological advancements reduce the unit cost of health care services, while at the same time increasing the demand for these services. The need to meet the health needs of the growing number of older people in the population is also an important issue. Due to limited capability for increased spending, new instruments are being sought to optimize the decision-making process in the health care sector. This, in itself, is difficult because it is hard to successfully implement change in the area of activities related to meeting the needs socially recognized as important and financed from public funds. In societies, a variety of emotions, views, attitudes, feelings, and interests remain in constant conflict with one another, and their incorrect identification can lead to the failure of even very well-prepared actions. Decisions on the directions and structure of fund allocation will determine whether or not the implementation of new solutions will actually contribute to the better functioning of the health care system and provide citizens with equal and more fair access to health care services, while at the same time the state will be able to control and measure the accomplishment of objectives.

The aim of this paper is to present the European Union's cohesion policy in the field of health care and to indicate the most important actions of implemented programmes/projects in the selected countries—The Visegrad Group—in the perspective of 2014–2020. The research methodology consists of analysis of scientific papers and documents as well as interpretation of indicated examples of actions undertaken by countries from The Visegrad Group financed from the EU cohesion policy funds.

## Milestones of the Cohesion Policy

The cohesion policy is the European Union's main investment policy, delivering benefits to all EU regions and cities and supporting economic growth, job creation, business competitiveness, sustainable development, environmental protection and health care. The EU's current cohesion policy is the result of decisions taken over the course of many years (7). When the European Communities were founded, major territorial and demographic differences existed that could have become obstacles to integration and development in Europe. The 1957 Treaty of Rome established solidarity mechanisms in the form of two funds: the European Social Fund (ESF) and the European Agricultural Guidance and Guarantee Fund (EAGGF, Guidance Section). The European Regional Fund (ERDF), set up in 1975, aimed to embrace regional issues. In 1994 the Cohesion Fund, was established. The signing of the Single European Act in 1986 marked the inclusion of economic and social cohesion within the Community's remit. In 2008, the Lisbon Treaty defined the third dimension of the EU's cohesion: territorial cohesion. These three dimensions of cohesion are supported by the cohesion policy and Structural Funds (8). Since 1988, the budget of the EU's cohesion policy has increased significantly, turning it into one of the most important EU policies, along with the Common Agricultural Policy. Cohesion policy reflects a new approach to health policy that takes into account impact on health determinants rather than just building a better health care system for people already suffering from health problems (9). The Cohesion Policy budget, which accounts for one third of the total EU budget (€351.8 billion in the period 2014–2020) (10), allows for the financing of many health-related investments, mainly toward the reduction of inequalities in key determinants of health (11). There is wide evidence that low income and low socioeconomic status are connected with poor health outcomes, particularly with respect to high mortality (12). By allocating funds to the regions where development is lagging behind, CP could act as a major driver of health equity (13).

The enlargement of the European Union in the 1980s had resulted not only in its increased territorial range, but it also caused varying levels of access to health care services. In 2004, the accession of Central and Eastern European countries (all the VG4 member states) created the need for further structural changes. Estimates relating to the enlargement indicated a 20% increase in population, accompanied by a mere 5% increase in total GDP (14). The Lisbon Treaty (2007) therefore emphasized strengthening economic, social, and territorial cohesion. The EU member states were obliged to prepare a national strategic reference framework defining strategic priority objectives and key areas of intervention within the cohesion policy. At present, a single set of rules embraces the five European Structural and Investment Funds (ESIF), connected with the Europe 2020 strategy. This aims to expedite smart, sustainable and inclusive growth in the EU, improve coordination, and ensure the coherent use of European Structural and Investment Funds and simplified access to funds for eligible entities. In 2013, the health care systems reflection process identified shared success factors for the efficient use of structural funds in health care. The analysis of the mapping of the use of European structural and investment funds in health provided the basis for planning funding for 2014–2020 and for the operational programmes of individual EU countries. The technical toolkit includes instruments and mechanisms, investment assessment, calls for proposals, indicators, and new health concepts and models. Projects under the European Structural and Investment Funds contribute to a variety of policy objectives such as: better access to healthcare, support to reform processes for efficient and resilient healthcare systems, use of digital/eHealth solutions, in particular those related to the Digital Single Market, and interoperability of such solutions across and between member states, research and innovation in health and life sciences, support to active and healthy aging, health and disease prevention, and actions in favor of health professionals (training, lifelong learning, employment planning, labor retention). In the current Europe 2020 strategy, cohesion policy is identified as the most important tool for initiating actions aimed at harmonizing social and economic living conditions in all regions of the EU member states. For the 2014–2020 period, a new legislative framework was adopted for the five main funds subject to the EU's cohesion policy, the common agricultural policy, and the common fisheries policy. These include the European Regional Development Fund (ERDF); the European Social Fund (ESF); the Cohesion Fund; the European Agricultural Fund for Rural Development (EAFRD); and the European Maritime and Fisheries Fund (EMFF) (15).

The implementation of one of the three basic priorities of the Europe 2020 strategy—inclusive growth, fostering a high-employment economy delivering social and territorial cohesion – in the countries benefiting from funds allocated under the cohesion policy requires allocating substantial financial resources for improved health care.

## Interventions in the Visegrad Group Countries Under the Cohesion Policy 2014–2020

The Visegrad Group (VG4) is a regional form of cooperation between four Central European countries: Poland, the Czech Republic, the Slovak Republic and Hungary, which share a variety of historical experiences, cultural traditions and values, as well as regional and similar geopolitical conditions (16). VG4 countries have a similar level of economic development, as confirmed by GDP per capita in US dollars: Poland (33,890), the Czech Republic (38,830), the Slovak Republic (36,640), Hungary (34,050) (17). Similarly, expenditure on health care is shaped both as a percentage of GDP and expressed as real expenditure in US dollars per capita—Poland (6.3%/2,056), the Czech Republic (7.5%/3,058), the Slovak Republic (6.7%/2,290) and Hungary (6.6%/2,047) (18). Since 2004, the VG4 countries have been members of the European Union, while the Visegrad Group is a forum for exchanging experiences and developing common opinions and official positions on issues of importance for the future of the region and the entire EU. Although the Visegrad Group countries have been using EU funds since 2004, they achieve significantly lower ratings in terms of outcomes within their health care systems. This result means that the functioning of a health care system depends not only on the applied model, but also on historical and economic determinants shaping the given health care system. In the VG 4 countries, the objectives of cohesion policy result from recognizing the increased competitiveness of national markets in the global economy, increased employment, and better standards of living would deter achieving a healthy population.

In the recent report, *Effectiveness of cohesion policy: Learning from the project characteristics that produce the best results 2019* (available at http://www.europarl.europa.eu/supporting-analyses, page 81-82) we found the statement that in the literature, “*there is no conclusion on the impact of cohesion policy. Macroeconomic simulations conclude that these funds have a positive effect, but such results are the reflection of the assumptions made. The results of empirical analyses have been mixed and inconclusive, suggesting that cohesion funds have the potential to generate significant growth, but do not always fulfill this potential.”* As there is no clear evidence for the positive impact of cohesion policy on macroeconomic development, we decided to indicate social impact as an assessment of the results of the impact of cohesion policy funds intended to support less developed EU countries, including the VG4. Social impact is the effect an organization's actions have on the well-being of the community.

We chose the EHCI ranking as an indirect source of information on social impact assessment of investment in health care.

The Health Consumer Powerhouse Ltd (HCP) was founded 2004, aiming to introduce open comparisons of healthcare systems performance as a tool to improve outcomes and to support patient and physician empowerment. Since then, HCP has published more than 50 editions of Health Consumer Indexes in several healthcare areas. We decided that indicators regarding the overall assessment of the health care system can confirm a positive relationship between the financing of actions under cohesion policy funds and the results achieved. The choice of the analyzed years is intentional. 2007 and 2013 are the dates of the beginning and end of new goals for cohesion policy and the financial resources allocated for them. 2018 is the last year for which data in the EHCI ranking is available. The analysis embraced the evaluation of the outcomes of the health care systems made by consumers/patients for the VG4 countries ranked according to the EHCI (European Health Consumer Index). EHCI is an attempt to measure and rank the effectiveness of health care service provision from the consumer perspective—literature also uses the term “patient-friendliness.” The quoted data confirm the statement about the positive impact of cohesion policy funds on the development of health care systems in the VG4 countries. Although the VG4 countries slightly change their position in the overall ranking, we note that the total sum of points awarded is increasing.

The VG4 countries benefit from the same EU programmes but adapt their use to their internal circumstances and needs. In the Czech Republic, the programmes which focused on employability, sustainable development, entrepreneurship, and innovation, as well as regional development and research, and education are the most important. Slovakia concentrates on human resources, infrastructure, regional development, and efficient public administration. In Poland, the majority of programmes are connected with infrastructure and environment, knowledge and education growth, and digital health. In Hungary, the emphasis is on human resources and territorial and settlement development, as well as economic development and innovation (see Table 1). The scope of the adopted intervention programmes in health care varies between the Visegrad countries. The greatest similarities are found between Poland and Slovakia, which carry out as many as 4 similar actions: a. improving access to primary and emergency care, b. prevention programmes for diseases that affect employees, c. access to affordable, sustainable, and high quality health services, and d. new developments in the formulation and implementation of public policies.

**Table 1:** The results achieved in EHCI in the Visegrad Group countries in the years 2007–2013, 2014–2018 and programmes, funding sources, and activities undertaken in achieving cohesion policy objectives in health care in the VG4.

**EHCI assessment, strategic goals and investment in financial perspective 2014–2020**			**Poland**	**Czech republic**	**Slovak republic**	**Hungary**
**Financial perspective** **2007–2013**	**2007**	Country position^*^	27	15	23	24
		Score^**^	447	612	532	513
	**2013**	**Country position**	33	15	21	28
		**score**	521	683	650	546
**Financial perspective** **2014–2020**	**2014**	**Country position**	33	15	20	25
		**score**	511	713	665	602
	**2018**	**Country position**	32	14	17	33
		**score**	585	731	722	565
**Strategic goals and sources of funding for health interventions 2014-2020**			[a] Operational Programme Infrastructure and Environment (sources: ERDF and CF) [b] Operational Programme Knowledge Education Growth (sources: ESF and Youth Employment Initiative) [c] OP Digital Poland (sources: ERDF) [d] ROP (sources: ERDF and ESF)	[a] Employment Operational Programme (source: ESF and YEI) [b] Operational Programme Prague - Pole of growth (source: ESF and ERDF) [c] Entrepreneurship and Innovation for Competitiveness Operational Programme (source: ERDF) [d] Integrated Regional Service Programme (source: ERDF) [e] Research, Development and Education Operational Programme (source: ESF and ERDF)	[a] Operational programme Human resources (sources: ERDF, ESF, CF and YEI) [b] Integrated Operational Programme Infrastructure (sources: ERDF, CF) [c] Integrated Regional Operational Programme (source: ERDF) [d] Efficient public administration under the operational programme (source: ESF)	[a] Human Resources Development Operational Programme (sources: ERDF and ESF) [b] Operational programme for territorial and settlement development (sources: ERDF and ESF) [c] Competing Operational Programme for Central Hungary (sources: ERDF and ESF) [d] Economic Development and Innovation Operational Programme (sources: ERDF, ESF and Youth Employment Initiative)
**Type of activity/ investment**			Improving access to primary care and emergency care [a]. Prevention programmes for diseases that affect employees [b]. Access to affordable, sustainable and high quality health services [b]. New developments in the formulation and implementation of public policies [b]. Improving the employability of vulnerable groups [b]. E-health [c.d] Types of investments under regional operational programmes [d]	E-health [a,c,d] Social care. Social services [d] Preventing social exclusion of people with social or medical problems [a,b,d,5]. Strengthening rescue services in disaster preparedness [e]	The transition to social services [a] Integration of care [c] Creating innovative clinical procedures [a] Promoting access to healthcare in marginalized communities [a] E-health [b] Strengthening institutional capacity [d] Human resource planning in health care[a]. Improving access to primary health care and emergency medical care [a]. Prevention programmes for diseases that adversely affect employees [b]. Access to affordable, sustainable and high quality health services [b]. - New developments in the formulation and implementation of public policies [b].	Increasing health awareness [a] Improving health services [a.b.c]. Social care and social services [c] Education and training of medical personnel [c] E-health [d] Health tourism [d]

* *Country position according to the place resulting from the number of points obtained in total score from 1 to 35*.

** *Maximum total score 1,000 points*.

*Source: own elaboration (19, 20)*.

All countries intervene in the field of e-health (see Table 1). They are mainly concerned with the improved effectiveness of public administration through the implementation of IT solutions and the digitisation of health operators and their resources, as well as improved ICT applications in the health care sector. The aim of e-health policy is to improve the interoperability of healthcare systems in order to provide easier access to information, reduce costs and improve performance within the system, as well as the introduction of telemedicine services on a larger scale and support for meeting European standards, interoperability testing, and certification of healthcare systems. Measures ensuring the development of access points to groups deprived of digital competences and vulnerable citizens, mainly in rural areas, are also important.

In the VG4 countries, important initiatives undertaken under the EU's cohesion policy concern the integration of social and health policies (priorities in the Czech Republic, Slovakia and Hungary). Actions in this area mainly consist of developing community support to help disadvantaged groups to enter the labor market, supporting the development of community centers, social housing and social enterprises, de-institutionalization of psychiatric care and development of community health care infrastructure (see Table 1).

## Conclusion

In microeconomic terms, good health is a prerequisite for financial success (analyzed at the individual and family level), while in macroeconomic terms, the well-being of society is an integral part of broadly defined human and social capital, which amounts to the unambiguous statement that the good health of society is an important determinant of economic growth (21). The growing significance of the health care sector, as one of the most dynamic areas of the labor market and—in the broader sense—an important sector of the economy, has caused health to have a strong presence in strategic documents constituting the foundation for the main directions of government policies (22). Poor health of the population has a number of economic consequences; above all, it is a significant burden on the economy. On the one hand, it causes the need to incur substantial public expenses related to treatment and social benefits; on the other hand, it leads to lower revenues and the impoverishment of the society (23). This is particularly important as regions in the analyzed countries are among the least developed regions in EU (with the exception of capital regions).

The member states are required to develop and implement plans, including investment priorities for the five European structural and investment funds. The agreements have been supplemented with recommendations prepared individually for each member state by the European Commission. Each country pursuing cohesion policy goals in the health care sector had to define and incorporate coordination mechanisms into public management.

As a result, the interventions conducted so far can be assessed as positively targeted and verified in the process of the actual implementation of cohesion programmes. The priorities and similar areas of activity in each analyzed country are e-health, population protection, and investment policy. In the coming years, it is also expected that, in line with the current EU policy, health related issues will be prioritized, which will be incorporated into specific policies, and then executive documents.

## Data Availability Statement

Publicly available datasets were analyzed in this study. This data can be found here: https://www.ewaluacja.gov.pl/strony/badania-i-analizy/wyniki-badan-ewaluacyjnych/badania-ewaluacyjne/ocena-mechanizmow-koordynacji-interwencji-publicznej-w-obszarze-zdrowia-stosowanych-w-ramach-polityki-spojnosci-w-polsce/, www.ec.europa.eu/health//sites/health/files/health_structural_funds, www.doi.org/10.1787/4dd50c09en.

## Author Contributions

TH and MW: original draft preparation, conceptualization, investigation and review, validation, and editing. IK-B: resources, investigation, results interpretation, validation, and editing. AF-W: methodology, investigation and review, formal analysis, validation, and editing.

## Conflict of Interest

The authors declare that the research was conducted in the absence of any commercial or financial relationships that could be construed as a potential conflict of interest.
